# New Hospital for Women

**Published:** 1902-03-15

**Authors:** 


### NEW HOSPITAL FOR WOMEN.

The annual meeting took place on Thursday, last week. The board-room was well filled, chiefly with women, and a good deal of disappointment was caused by the announcement that Adeline, Duchess of Bedford, who was expected to preside, was too ill to come. The Rev. H. L. Paget, vicar of St. Pancras, took her place, and urged the claims of the hospital on three special grounds: (1) the great increase in the numbers applying for out-relief; (2) the need for a children's department; and (3) the want of the present moment, an endowment for the pathological department.

Colonel Montefiore, who has been secretary of the Medical Committee of the Charity Organisation Society for some years, spoke of the valuable work that might be done by an almoner in the out-patients' department; the plan worked well at the Royal Free, where the numbers, so far from diminishing as a result of the inquiries made into the circumstances of the patients, had increased since the appointment of the almoner.

### Endowment for Pathology.

Mrs. Stanley Boyd, M.D., said that the vacant place on the committee, caused by Mrs. Webb's much regretted retirement, would be taken by Miss Chadwick, whose beautiful and accurate microscopical drawings were of such value in the pathological work of the hospital. This department had sustained a great loss in the retirement, through professional engagements, of Mrs. Fleming, M.D., whose work had been voluntary, and now the committee felt very strongly that something must be done by way of an endowment, for a competent pathologist must be found, and unfortunately she had to live. It was not, in a financial sense, paying work. The hospitals ought to contribute to the sum total of the knowledge of disease, and, as a consequence, its better treatment. It was to the pathological department that the medical world looked for light and guidance, and the importance of it, both to the present generation and to future ones, was very great. Not only whether an operation should take place at all, but what it should be, and to what extent it should go—were questions that could only be settled by increased pathological knowledge. Valuable as the work was, it was not easy to find another woman with the leisure and the means to devote to it without remuneration. The committee had given it their consideration, as they did whenever the medical staff made a suggestion, and they were willing to devote what funds they felt the hospital could spare; Mr. Chadburn had also given a large special donation. But even that was not sufficient for the amount of time the staff wanted given to the work, and an endowment was greatly needed if this hospital was to take its place among others in the study, as well as the treatment, of disease.

Mrs. Garrett Anderson, M.D., speaking immediately after Mrs. Boyd, said that she had had a letter a few hours before the meeting began, with the welcome news that £13,000 had been given for the very object for which Mrs. Boyd had been pleading. The speaker meant to hold it tight, and invest it at an interest of £500 a year for the Pathological Department. She believed in having a good character for finance, and she did not hold at all with falling into debt. She proposed investing it in the War Loan.

### Medical Women in America.

Mrs. Garrett Anderson went on to speak of a statement that had been going the round of the papers to the effect that the medical schools for women in America were being closed. She had taken the trouble to investigate the matter, and found that it was untrue. The facts were these: The North-West University tried to keep up two medical schools, and naturally did not succeed. There were over 80 schools that received medical women in America, and several others had recently opened their doors; so that it was not in the least surprising that this poor unendowed school, taken over by the University, had to be closed. There was nothing in the romance after all. Moreover, in four hospitals in Chicago all the resident staff were women ; and in seven, women were working on the permanent staff, together with men. At Rush and at Chicago women were teaching men students. That did not look like failure. "That lie," Mrs. Anderson concluded, "has been going round through the world, and it is well to stop it."

The report was adopted, and the usual votes of thanks ended the meeting.

